# Patient reported outcome of 33 operatively treated talar fractures

**DOI:** 10.1186/s12891-021-04572-3

**Published:** 2021-08-16

**Authors:** Patrick Pflüger, Michael Zyskowski, Anne Weber, Katharina Gleisenberg, Chlodwig Kirchhoff, Peter Biberthaler, Moritz Crönlein

**Affiliations:** Department of Trauma Surgery, Klinikum rechts der Isar, Technical University of Munich, Ismaninger Str. 22, 81675 Munich, Germany

**Keywords:** Talar fractures, Patient reported outcome, ORIF, Follow-up study

## Abstract

**Background:**

Management of talar fractures remains to be one of the most challenging aspects in trauma surgery. Unfortunately, the evidence regarding the correct treatment of these fractures is mainly based on retrospective case series, while studies assessing the patient-reported outcome are rare. Therefore, the aim of this trial was to analyze the patient reported outcome in context of trauma mechanism and concomitant injuries following operative treatment of talar fractures.

**Methods:**

A retrospective outcome study of patients with operatively treated talar fractures between 2003 and 2015 was conducted. The fractures were classified according to AO-/Hawkins classification system and to the Marti-Weber classification. Data was collected via patient registry, radiographs and a validated patient-reported outcome measure (PROM) for foot and ankle pathologies (Foot and Ankle Outcome Score = FOAS). An analysis regarding the functional outcome, concomitant injury and timing of surgery using the nonparametric Mann-Whitney U test and Spearman`s rank correlation was performed.

**Results:**

In total the functional outcome of 32 patients suffering from fractures to the talus were analyzed. The median age of the study cohort was 35±12.2 years, including 9 female (28 %) and 23 male (72 %) patients. The median FAOS score was 72±22.7 (range 13–94). Patients with an isolated talar fracture had an FAOS of 87±20 and with concomitant injury a score of 60±23.4 (*p* = 0.016). Patients with a closed talar fracture without emergency operation due to dislocation or polytrauma, showed no correlation between timing of surgery and FAOS (*r*= -0.17, *p* = 0.43). 10 % of the patients developed an avascular necrosis and 25 % showed signs of a posttraumatic arthritis. The follow-up time was 41 months (range: 16–145).

**Conclusions:**

Talar fractures were typically caused by high-energy trauma often associated with additional injuries of the lower extremity. The majority of the patients showed a fair to poor functional long-term outcome. Concomitant injuries of the lower extremity led to a lower FAOS. In closed talar fractures without the necessity of an emergency surgical intervention, time to surgery did not influence the patient reported outcome. Relating to the presented data, delayed surgery after soft tissue consolidation was not associated with a higher risk of developing an avascular necrosis.

## Introduction

The incidence rates of foot fractures amount to 140–226/100.000 per year, accounting for about 40 % of all fractures of the lower extremity [1–3]. However, only less then 10 % of all foot fractures involve the hind foot and the incidence of talar fractures is rare [1]. Typically, talar injuries are seen in men under the age of 40 years following a high-energy trauma [1, 4, 5]. Due to the high-energy mechanism, the rates of associated fractures are high [5]. Despite their rare incidence, talar fractures present a relevant and challenging injury for trauma surgeons by causing permanent disability of the ankle and foot function [6, 7].

Anatomically, the talus can be divided into 3 main structures: the body, the neck and the head. The talus has a unique position by forming the connection between lower thigh and foot. Its surface is mainly covered by articular cartilage without any tendon insertions and the blood supply is provided by a rich network of extra- and intraosseous anastomoses [8]. Due to these special anatomical features, avascular necrosis (AVN) and post-traumatic arthritis (PTA) are frequent complications following traumatic talar fractures [4, 6–8]. Reviews report osteonecrosis rates of up to 33 % and post-traumatic arthritis rates up to 81 % [4, 9]. In the last decade the rate of osteonecrosis and post-traumatic arthritis decreased, but still remains high and is associated with a poor long-term outcome [7, 9].

The most frequent used classification system for talar neck fractures was developed by L.G. Hawkins in 1970 in a case series of fifty-five patients with vertical fractures of the neck of the talus and later modified by Canale [10, 11]. The revised AO fracture and dislocation compendium of 2018 adapted the Hawkins classification system [12]. Talar body fractures can be classified according to Marti-Weber [13].

Due to their infrequent occurrence the level of evidence regarding the management of talar fractures is predominantly limited to case series [4, 6, 9].

To the authors’ knowledge, only three studies have been published since the year 2000, that reported the outcome following operative treatment of talar fractures with a validated patient-reported outcome measure (PROM) for foot and ankle pathologies [7, 14, 15]. Two of them only included talar neck fractures [14, 15].

Therefore, the aim of the presented study was to analyze the patient reported outcome in the context of trauma mechanism and concomitant injuries following operative treatment of talar fractures.

## Methods

### Patients

The study was approved by the institutional ethics board (No: 409/15 S, Technical University of Munich) and performed in accordance with the ethical standards of the 1964 Declaration of Helsinki as revised in 2000 and those of Good Clinical Practice. This retrospective case series study was conducted between 2005 and 2015 in our level I trauma center. All patients presenting with a talar fracture at our department of trauma and orthopedic surgery were reviewed for enrollment. Patients > 16 years, who were capable of giving informed consent and underwent operatively management were enrolled. Specific patient exclusion criteria included pathological fractures, substance abuse, presenting for revision surgery after external operation and patients with a legal guardian.

The electronic database of the hospital was retrospectively searched for patient characteristics. General data such as age at the time of injury, gender, trauma characteristics (affected side, mechanism of injury, concomitant injuries), treatment characteristics (surgical delay, duration of surgery, hospitalization), rate and type of revision surgery as well as date of latest follow-up were collected.

### Surgical technique

A board certified trauma surgeon, specialized in lower extremity surgery evaluated indications for surgery. Definite criteria for operative management were an open fracture and a dislocation of the ankle joint with unsuccessful closed reduction. Due to the importance of the talus for the ankle joint, conservative treatment is only limited to nondisplaced fractures or to patients with multiple comorbidities not able to endure surgery/anaesthesia. All the other patients with displaced fractures were indicated for operative treatment. Operative treatment involved temporary external fixation in case of major dislocation, open reduction and internal fixation (ORIF) with screw-, plate- or K-wire osteosynthesis. In case of a closed talar fracture, without dislocation in the talocrural, talocalcaneonavicular and subtalar joint and therefore a low risk of developing avascular necrosis, surgery was performed after soft tissue consolidation. The patient was placed in a supine position, prophylactic cefuroxime 1.5 g was administered and a thigh tourniquet applied and inflated in case to control bleeding. Usually in talar neck and body fractures preliminary fixation was achieved with the help of K-wires and after radiographic control definitely secured with cannulated screws. In case of comminuted talar body fractures, where screw osteosynthesis was not suitable, plate osteosynthesis was performed via an anteromedial or anterolateral approach. Postoperative aftercare included partial weight-bearing with 15 kg for 6 weeks in a walking boot using two crutches and anti-thrombotic therapy until full weight bearing.

### Patient reported outcome

All patients, who met inclusion criteria were contacted by mail to provide written informed consent. To quantify the patient reported outcome, the Foot and Ankle Outcome Score (FAOS) was used. The FAOS is a self-administered patient-relevant outcome questionnaire consisting of 42 items (range: 0-100) and includes items for activities of daily living, sport and recreational activities as well as foot- and ankle-related quality of life items. The German version of the FAOS is a valid and reliable instrument for foot and ankle patients [16]. The FAOS results were graded in “excellent” (100 − 90), “good” (89 − 80), “fair” (79 − 70) and “poor” (< 70) adapted from the evaluation of Thordarson et al. [17]. After hospital treatment and standard postoperative visits, patients were invited by mail to complete the FAOS. Only patients with a minimum follow-up of 12 months were included for further analysis.

### Radiographic outcome

Radiographic data was extracted of the institutional Picture Archiving and Communication System (PACS) and conventional radiographs in the preoperative, postoperative and follow-up periods and preoperative CT scans were analyzed. Talar fractures were classified based on radiographs using the AO classification system [12] and the Marti-Weber classification [13]. The standard outpatient aftercare in our clinic involves visits to our ambulatory facility with radiographic follow ups 6 weeks, 3 months and 1 year after trauma.

#### Statistics

Data was presented as median ± standard deviation (SD) or range (min; max). RStudio (RStudio Team (2020). RStudio: Integrated Development Environment for R. RStudio, PBC, Boston, MA URL http://www.rstudio.com/) was used for data processing.

Shapiro-Wilk test was performed to test for normality. The nonparametric Mann-Whitney U test was used to assess significant differences between two groups. In case of more than two groups the Kruskal-Wallis test was performed. To calculate the strength and association of two categorial variables, we calculated the odds ratio and 95 % confidence interval. The correlation between two continuous variables was assessed with Spearman correlation. P-value < 0.05 was considered statistically significant.

## Results

### Patients data

In total, 45 patients were treated at our level I trauma center with a fracture of the talus.  Thirty-five patients underwent surgery while 10 were treated conservatively. According to the inclusion criteria, 32 patients with 33 fractures were available for further data analysis. The median age of the study cohort was 35±12.2 years, including 9 female (28 %) and 23 male (72 %) patients. Female patients had an average age of 34±13.5 years and male patients of 36±11.8 years. In 14 patients (44 %) the right side was injured, whereas 17 (53 %) patients fractured their left and one patient both sides (3 %). The patients’ details are summarized in Table 1.

**Table 1:** Demographic, clinical and radiological characteristics

**Demographics**	Age, y, median, ±SD	35±12.2
	Gender	• 9 female (28%) • 23 male (72%)
	ASA score	• 1: 25 (78%) • 2: 6 (19%) • 3: 0 • 4: 1 (3%)
**Fracture type**	Body fracture	22 (69%)
	Neck fracture	7 (22%)
	Head fracture	3 (9%)
**Trauma mechanisms**^**a**^	Ankle distortion during sports	9 (28%)
	Road traffic accident	7 (22%)
	Fall <3m during sports	4 (13%)
	Fall <3m	4 (13%)
	Ankle distortion	2 (6%)
	Fall > 3m during sports	2 (6%)
	Fall >3m	1 (3%)
	Crush injury of the foot	1 (3%)
**Associated injury lower extremity**		15 (47%)
**Timing to surgery**		5.5 days (range: 0-19)
**Type of definitive treatment**	SOS	23 (72%)
	POS	5 (16%)
	K-Wiring	1 (3%)
	SOS+K-Wiring	1 (3%)
	Primary arthrodesis	1 (3%)
	Resection of the lateral process	1 (3%)
**Reintervention rate after definite operation**^**b**^	Implant removal	12 (38%)
	Superficial infection	2 (6%)
	Deep infection	1 (3%)
	Malreduction	1 (3%)

*SOS* screw osteosynthesis, *POS* plate osteosynthesis

^a^ In two cases (6%) there was no further information on the trauma mechanism

^b^ Implants were removed due to infection during revision surgery in two patients, the K-wires after two months (*n*=2) and in eight patients due to bone consolidation

The most common trauma mechanisms were an ankle distortion during sports in nine patients  and 7 patients were injured during a road traffic accidents. In two cases (6 %) there was no further information regarding the trauma mechanism (Table 1). According to the ASA (American Society of Anaesthesiologists) classification, 25 patients were classified as ASA 1 (78 %), six as ASA 2 (19 %) and one patient as ASA 4 (3 %). Concomitant injuries of the lower extremity were observed in 15 patients (47 %, Table 2). One patient had an open talar fracture and presented as a polytrauma following a road traffic accident. In total nine patients had a dislocation in the talocrural or talocalcaneonavicular and subtalar joint.

**Table 2:** Type of associated injury, frequency and percentage

Type of associated injury	Frequency	Percentage
Midfoot fractures	5	33%
Distal fibula fracture	3	20%
Lisfranc joint luxation	2	13%
Metatarsal fracture	1	7%
Distal tibia and fibula fracture	1	7%
Distal tibia fracture	1	7%
Luxation of the ankle joint with severe injury of the ligamentous apparatus	1	7%
Chopart luxation	1	7%

### Treatment

Dislocated fractures were treated with immediate open reduction and internal fixation in five cases, 3x with temporary ankle spanning external fixation and in two patients with a successful closed reduction followed by ORIF. Time between trauma and surgery was 5.5±5.4 days (range: 0–19). The duration of surgery was 82 min (range 45–191). Cannulated screw osteosynthesis was the most frequent performed operation in 23 patients (72 %), followed by plate osteosynthesis in five patients (16 %), K-wires and screw osteosynthesis in one patient, solely K-wire osteosynthesis in another patient and one time an open resection of the lateral process of the talus was performed. One patient, where reconstruction was not possible due to massive comminution, underwent tibio-talo-calcaneal (TTC) nailing. In total, four patients (13 %) needed revision surgery. Reasons were postoperative infection (*n* = 2), malreduction (*n* = 1) and superficial wound infection after implant removal (*n* = 1). Implants were removed due to infection during revision surgery in two patients, the K-wires after two months (*n* = 2) and in eight patients due to bone consolidation after 16±3 months.

### Radiological outcome

Preoperative CT scans were available for 30 patients and the talar fractures were classified according to the classification systems described. There were 22 body (69 %), 7 neck (22 %) and 3 head (9 %) fractures. The talar body fractures could be subclassified according to the AO classification system in 7 type A, 10 type B and 5 type C fractures. Amongst the talar neck fractures there were 6 type A (Hawkins 1) and 1 type C (Hawkins 3) fracture. The talar head fractures were classified in two patients as type B and in one patient as type C. Following the classification system of Marti-Weber, there were 14 Type I, 6 Type II, 8 Type III and 4 Type IV fractures. Patients with a minimum radiographic follow-up of 6 months (*n* = 20) showed an avascular necrosis in 2 cases (10 %, one type C talar body and one Hawkins type 3 talar neck fracture) with signs of PTA and 3 patients solely a PTA (PTA rate total: 25 %). Patients with an AVN underwent definite surgery on the same day of trauma and 19 days after injury. No patient developed a nonunion. The median follow-up time was 17±23.2 months (range: 6–97).

### Functional outcome

**Fig. 1:**
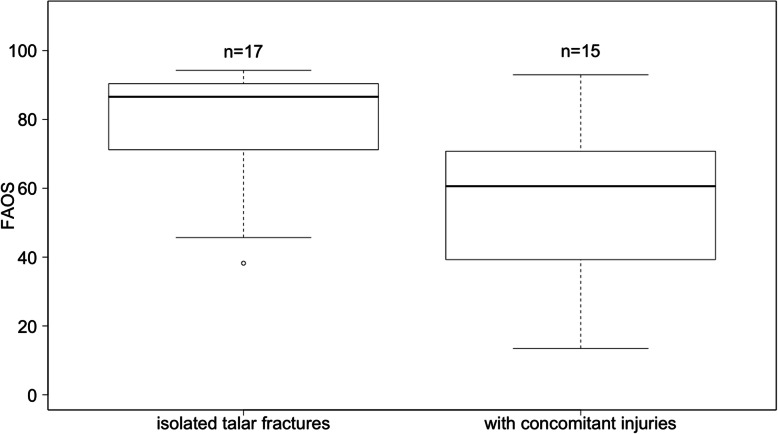
Boxplots of the FAOS score in talar fractures divided into isolated talar fractures and with concomitant injuries. Whiskers showing minimum and maximum. N = Number of patients

The median FAOS score was 72±22.7 (range 13–94). 7 patients (22 %) showed an excellent (100 − 90), 6 patients (19 %) a good (89 − 80), 4 patients (12 %) a fair (79 − 70) and 15 patients (47 %) a poor (< 70) result. Overall, patients reported the lowest scores regarding the items for symptoms and foot- and ankle-related quality of life. Patients with an isolated talar fracture had an FAOS of 87±20 and with concomitant injury a score of 60±23.4 (*p* = 0.016) (Fig. 1). A concomitant injury of the ipsilateral lower extremity was 10.67 (95 % KI: 1.10; 103.15) more likely for patients suffering from a road traffic accident. Patients with a closed talar fracture without emergency operation due to dislocation or polytrauma, showed no correlation between timing of surgery and FAOS (*r*= -0.17, *p* = 0.43). The FAOS score for Marti-Weber type I fractures was 73±22.8, type II 80±17.9, type III 66±28.6 and type IV 60±20.2 (*p* = 0.71). The follow-up time was 41 months (range 16–145).

## Discussion

The management of talar fractures remains to be one of the most challenging aspects in modern trauma surgery, often leading to a poor functional outcome for the patient [5, 9, 18]. The understanding of the unique anatomy and its role for force transmission in the ankle joint is crucial for a successful treatment [5]. The evidence regarding the treatment of talar fractures is mainly based on retrospective case series [6, 9]. Clinical trials assessing the patient-reported outcome are rare [7, 14, 15]. Therefore, the aim of the presented study was to analyze the patients` reported outcome following operative treatment of talar fractures. In general, there is a slight trend towards patient reported outcome measurement tools, since they provide benefits regarding safety, effectiveness and experience (28). Patients become involved by directly assessing their symptoms, disability and quality of live, thereby avoiding observer bias.

Overall, the functional outcome of 32 patients suffering from talar fractures treated between 2003 and 2015 was assessed. The median age of the study cohort was 35 years and the majority of the patients were male (72 %). Epidemiological studies and reviews of talar fractures also demonstrated a similar age and gender distribution with peak incidences in men under the age of 40 years [1, 18]. Considering the ASA classification, the vast majority presented without systemic organic pathologies, which is in line with other current published studies on talar fractures [7, 19]. Ankle distortions during sports (28 %) and road traffic accidents (22 %) emerged to be the most common causes for talar fractures in the patient collective. Most notably due to the associated high force impact to the ankle joint, concomitant injuries were frequently observed [5, 6, 19, 20]. Patients suffering from a road traffic accident were more likely to sustain a concomitant injury of the ipsilateral lower extremity. In a recent study Vints et al. reported an occurrence of associated fractures of the same foot or ankle of 60 % [7]. In the presented study, a concomitant injury of the lower extremity was observed in 47 % of the patients resulting in a poorer FAOS score.

The median FAOS score was 72 and over the half of patients showed a fair to poor result, especially regarding the categories symptoms and quality of life. This is in line with a current study of Vints et al. reporting, that talar fractures have a major impact on physical health status measured by the SF36 score [7]. Biz et al. stated, that 60 % of their patients showed a good-excellent AOFAS following ORIF of isolated talar neck and body fractures [19]. The on average a bit inferior outcome might be due to  the study design, using a solely patient reported outcome measurement tool. In contrast to the AOFAS, the FAOS score includes items for activities of daily living, sport and recreational activities and foot- and ankle-related quality of life items. Due to the severity of the injury low self-reported ratings in these categories are plausible, leading to an overall lower score.

Regarding the surgical treatment the most frequent performed operation was screw osteosynthesis in 72 % of the patients. This is the predominant method of fixation for talar neck fractures and widely used. In the last decade plate osteosynthesis has become more popular, especially in case of comminuted talar fractures [5, 8].

The timing of surgery is still disputed, since historically immediate operative treatment was recommended to reduce the risk of osteonecrosis with talar neck fractures [5, 21]. In the investigated study, timing of surgery did not influence the FAOS of patients with a closed talar fracture. Furthermore, there was no higher risk for an AVN in patients undergoing delayed surgery after soft tissue consolidation. This is in line with Dodd A. et al., who analyzed the outcome following talar neck fractures and found no correlation between timing of surgery and rates of osteonecrosis [9]. A recent retrospective study of 31 operatively treated patients with talar neck and body fractures, also reported, that the operation timing did not influence AVN development and clinical outcome [19]. The recommendation for the time of fixation of closed talar fractures has changed and the consideration of soft tissue complications as well as surgical experience became more relevant [5, 7].

The revision rate after definite surgery was low and only three patients needed additional surgery due to a malreduction and postoperative wound infections. Vints et al. reported a postoperative site infection rate following operative treatment of talar fractures of 4 % [7]. Biz et al. stated a wound infection rate of 3.6 % and a malunion rate of 21.4 % after operative management of isolated talar neck and body fractures, but none of their patients needed revision surgery due to the complications [19].

The radiological data analysis showed an AVN in 10 % of our patients and a PTA rate of 25 % after a follow-up time of 17 months. The AVN rates, reported in the current literature, vary depending on the fracture type, the soft tissue involvement and the period of time the study was conducted [5]. A systematic review of 340 patients with talar neck fractures reported an overall AVN incidence rate of 26 % [18]. The stratified analysis revealed, that the AVN incidence rates were significantly higher in Hawkins type III and IV talar neck fractures [18]. The lower AVN rate might be explained by the fact, that the presented study included 69 % talar body fractures. Talar body fractures show lower AVN rates and thus this might be a possible explanation for the findings [15]. In contrast to the literature, the reported PTA rate is considerably lower . A recently conducted study stated a PTA rate of 81.4 % after a follow-up time of 7 years [19], while another current study found an osteoarthritis rate of 42 % after a follow-up of 9 years [7]. A meta-analysis by Dodd A. et al. investigating the outcome of talar neck fractures, calculated a mean PTA rate of 49 % for 16 included studies [9]. The reported rates ranged between 4% to 100 % and studies with a follow-up time less than two years showed the lowest PTA incidence rates [9].

The results of our study may be influenced by several potential limitations. Due to its retrospective design, there was no randomization of the patients. Further, long-term clinical and radiological results were not part of our study and radiographs were taken for clinical follow-up and not for research purposes in a strict standardized manner. The surgical procedures evaluated in this study were performed between 2003 and 2015 and especially in regards to the first years the type of implants and surgical management evolved over the years. The FAOS is a validated self-reported functional outcome questionnaire for foot and ankle pathologies, but there is no validation study in patients with talar fractures. Despite strong validity and correlation with other common self-reported outcome scores, this needs to be considered by interpreting the reported functional outcome.

However, to the authors’ knowledge, our study differs from the previous published studies by providing current evidence regarding the patient-reported outcome following operative treatment of fractures to the talus.

Talar fractures are severe injuries of the foot, typically caused by high-energy trauma and often associated with additional injuries of the lower extremity. Considering the complexity of this fracture entity, the functional long term-outcome can be estimated as fair to poor. Concomitant injuries of the lower extremity led to signficantly lower FAOS. In closed talar fractures without the necessity of an emergency surgical intervention, time to surgery did not influence the patient reported outcome. Relating to the presented data, delayed surgery after soft tissue consolidation was not associated with a higher risk for an AVN.

## Data Availability

The datasets generated during and analyzed during the current study are not publicly available due to them containing information that could compromise research participant privacy/consent, but are available from the corresponding author on reasonable request.

## Abbreviations

AVN: Avascular necrosis
FOAS: Foot and Ankle Outcome Score
ORIF: Open reduction and internal fixation
PTA: Post-traumatic arthritis
PROM: Patient-reported outcome measure
